# Are Cooked Nutritious School Lunches Associated with Improved Attendance? Findings from the 2022–2023 Tasmanian School Lunch Project

**DOI:** 10.3390/nu16193393

**Published:** 2024-10-06

**Authors:** Kylie J. Smith, Verity Cleland, Kate Chappell, Brooklyn Fraser, Laura Sutton, Fiona Proudfoot, Julie Dunbabin, Kim Jose

**Affiliations:** 1 Menzies Institute for Medical Research, University of Tasmania, Hobart, TAS 7000, Australia; verity.cleland@utas.edu.au (V.C.); kate.chappell@utas.edu.au (K.C.); brooklyn.fraser@utas.edu.au (B.F.); laura.sutton@utas.edu.au (L.S.); fiona.proudfoot@utas.edu.au (F.P.); kim.jose@utas.edu.au (K.J.); 2 Alliance for Research in Exercise, Nutrition and Activity (ARENA), Allied Health and Human Performance, University of South Australia, Adelaide, SA 5000, Australia; 3 School Food Matters, Hobart, TAS 7004, Australia; julie@schoolfoodmatters.org.au

**Keywords:** attendance, school meals, lunch, nutrition, cooked lunches, students, schools

## Abstract

**Background/Objectives:** During 2022–2023, the School Lunch Project (SLP) provided free nutritious cooked lunches 1–4 days per week to Kinder to Grade 10 students attending 30 schools in areas of high disadvantage in Tasmania, Australia. This analysis examined if the SLP was associated with student attendance. **Methods**: Staff (teachers, support staff, and principals) from 12 schools completed an online survey and/or participated in focus groups/interviews. Government-held, objectively measured, grade-level attendance data were provided for 17 SLP and 11 matched comparison schools for 2018–2023. Linear mixed models compared attendance on school lunch and non-school lunch days in SLP schools. Difference-in-difference regression compared attendance between SLP and comparison schools. Qualitative data were analysed thematically. **Results**: Sixty-five staff completed surveys, where 22% reported that increased attendance was a benefit of the SLP. Similar findings were observed in the staff focus groups/interviews (N = 51). Mean attendance was similar on school lunch and non-school lunch days among the SLP schools during 2022 (difference: 0.04, 95% CI: −0.5, 0.6) and 2023 (difference 0.1, 95% CI: −0.2, 0.4) and similar between SLP and comparison schools (average treatment effect in the treated: 1.2, 95% CI: −0.7, 3.0). **Conclusions**: The SLP was perceived by some staff to improve attendance but was not associated with objectively measured attendance examined at the grade level.

## 1. Introduction

Universal school meal programs, which provide breakfast and/or lunch to all students regardless of their family circumstances, can increase food security and improve student diet quality [1]. Free school meals may also increase school attendance if students from food-insecure households attend school to access food, and improved diet quality may improve health and reduce absences due to illness [2,3]. A theoretical cost–benefit study found that improving diet quality was a cost-effective way to improve attendance among students with poor diets [4]. Increasing school attendance is a key priority in education policy, as low attendance has been associated with poor academic performance [5,6].

Studies examining associations between universal school lunches and attendance have reported inconsistent findings. A 2022 review by Cohen et al. included five studies that examined associations between daily school lunch programs and attendance; one study found increased attendance among elementary but not middle school students, and another study found a decrease in the percentage of economically disadvantaged elementary students with low attendance (absent at least 5% of school days) in the second year of implementation. The three remaining studies found no association between lunches and attendance; however, two of those studies only provided daily lunches for up to three months [3], and one study only included 17 students [7].

Since that review, the New Zealand Government introduced a school lunch program, Ka Ora Ka Oka, which provided daily lunches to students attending schools in areas with high levels of deprivation. The school lunches were not associated with overall attendance [8]. However, secondary analysis found attendance increased one to two days per term among the most underserved students (those who would rarely have enough food to eat without school lunches) [9]. The School Nutrition Project, in the Northern Territory of Australia, provides daily lunch plus combinations of breakfast, morning tea, and afternoon tea in 73 schools in remote and very remote communities [10]. Key stakeholders reported increased attendance during the project, but objective attendance data were not collected. In Scotland, daily universal free school lunches for students in Kinder to Grade 3 had little impact on absences [11]. A 2024 review, which focused on longitudinal studies in the USA, did not include any studies additional to the Cohen review [12].

Australia does not have a national school lunch program, with most students taking a packed lunch from home or buying from a school canteen [13]. Foods that are included in school lunch boxes and items most frequently purchased from school canteens are often inconsistent with dietary guidelines [14,15]. In addition, some children come to school without food or do not have enough food to eat due to food insecurity. Food insecurity is becoming more prevalent in Australia, with one-third of Australian households with children estimated to be food insecure in 2022 [16]. Similar findings were reported in a recent Tasmanian survey, where 28% of households with children reported some food insecurity for their children [17]. To improve diet quality and ensure children have at least one nutritious meal each day, there is increasing interest in developing school lunch programs in Australia [18,19].

During 2022–2023, the Tasmanian state government funded the Tasmanian School Lunch Project, which provided nutritious cooked lunches one to four days per week in 30 schools in areas of high socioeconomic disadvantage [20]. The Project was delivered by School Food Matters, a not-for-profit health promotion organisation, and built on a previous feasibility study conducted in three Tasmanian schools over four weeks [21].

This study examines whether the School Lunch Project was associated with staff-perceived attendance and routinely collected attendance data. The three key objectives were to (1) examine perceptions of change in student attendance among school staff in the School Lunch Project schools, (2) compare attendance on the days the lunches were provided to days the lunches were not provided among the participating School Lunch Project schools, and (3) compare overall attendance in the School Lunch Project schools to schools that were not participating in the School Lunch Project.

## 2. Materials and Methods

### 2.1. Tasmanian Schools

In Tasmania, the school year usually begins in January/February and ends in December. The year is divided into four terms of approximately 10 weeks each. Primary schools are for Kinder to Grade 6 students (approximately 4 to 12 years old), secondary schools are for Grade 7 to 10 students (12–16 years old), and college is for Grades 11–12 (17–18 years old; not included in this study). District schools can include primary, secondary, and college students.

### 2.2. School Lunch Project Schools

During July 2021, all 197 government schools (equivalent to public schools in the USA) with Kinder to Grade 10 students were invited to submit an expression of interest to participate in the School Lunch Project. Fifteen schools were selected. The project was planned to start in term 1, 2022, but due to the high number of COVID-19 cases, the Department for Education, Children and Young People introduced a moratorium on new projects, including research, in term 1 so that schools could focus on learning. Therefore, the School Lunch Project commenced in term 2, 2022. The expression of interest process was repeated in 2022, with 15 schools selected to commence in term 1, 2023. The successful schools were all from areas of high disadvantage. The lunches aligned with the 2013 Australian Dietary Guidelines [22]. The menus and recipes were designed by dietitians, with input from chefs and School Food Matters staff [23]. The School Lunch Project was intended to provide daily lunches to all students. However, this was not possible due to limited funding and resources. Each school decided how many days per week the lunches would be provided and whether they would provide lunches for the whole school or selected grades/classes. The lunches were available to all students in the selected grades/classes. Parental consent was required for the students to receive the lunches.

### 2.3. Comparison Schools

Comparison schools were not involved in the School Lunch Project and were used to help determine if any observed changes in attendance were due to the School Lunch Project rather than other initiatives the schools and government had introduced to increase attendance. A publicly available website [24] was used to select government schools of the same school type (primary, secondary, and district) with similar numbers of enrolled students and levels of disadvantage to the School Lunch Project schools. The Index of Community Socio-Educational Advantage (ICSEA) percentile was used as the measure of disadvantage. ICSEA is calculated using student characteristics (parent’s occupation and parent’s education), the geographical location of the school, and the proportion of Indigenous students [25]. As an example, an ICSEA percentile of 8 indicates the school is more educationally disadvantaged than 92% of Australian schools.

### 2.4. School Lunch Project Evaluation

This research is part of a larger evaluation of the School Lunch Project. A developmental evaluation approach was used, which supports social innovation, adaptive management, and systems change and allows real-time feedback to the implementors of the program [26]. This research was approved by the University of Tasmania Human Research Ethics Committee (ID:26744, 14 December 2021) and the Tasmanian Department for Education Children and Young People (Education and Performance Review Committee, FILE 2021-47, 14 December 2021).

Each year, six School Lunch Project schools were selected to participate in the evaluation of the project [20]. Principals were approached to provide consent for their school (all agreed to participate) and help facilitate communication with their school community. Informed consent was obtained from the school staff for their participation.

### 2.5. Surveys and Focus Groups

Schools were asked to distribute information about the research project to staff via their preferred communication channel. School staff (teachers, support staff and principals) were invited to complete an online survey and/or participate in a focus group or interview during term 3 or 4, the year their school started providing the lunches. Amongst other items about the school lunches, the survey asked, ‘Have you noticed any changes in attendance among participating students since the school started providing lunches?’ The response options were ‘No’ or ‘Yes’. When the response was ‘yes’, staff were asked to ‘please describe what the changes are and what you think may explain these changes’ (open-ended text response). The survey also asked, ‘What do you think are the benefits of the lunches?’; staff could choose multiple options, including ‘Improved school attendance’. Focus groups and interviews were held at each school by trained facilitators and were audio-recorded and transcribed verbatim. The focus groups covered a broad range of topics, reflecting the wide scope of the School Lunch Project (for example, assessing the feasibility and acceptability of providing cooked lunches in Australian schools). Thematic analysis was conducted using qualitative data management software NVivo 12 (release 1.6.1, 2022, Lumivero, Denver, CO, USA), and the results relating to attendance are presented.

### 2.6. Routinely Collected (Objectively Collected) Attendance Data

The principals of all 30 School Lunch Project schools and 30 comparison schools were invited by email to consent for their school’s routinely collected (objectively measured) government-held student attendance data to be provided to the research team. Schools that had not consented after two weeks were contacted by phone, and a final reminder email was also sent. The completed consent forms were emailed to the Tasmanian Department for Education, Children and Young People, who compiled the attendance dataset.

Daily attendance rates for each school were provided by grade from 7 February 2018 to 30 November 2023 (inclusive). Four years of baseline data (2018–2021) were used to reduce the potential impact COVID-19 had on attendance in the two years prior to the project (2020–2021). The attendance rates were calculated as minutes present (sum of the number of minutes for all students marked as ‘present’ for timetabled periods in each grade) divided by total minutes (sum of the number of minutes each student was timetabled to attend during that day for each grade).

To reduce the potential to identify individual students, the Tasmanian Department for Education, Children and Young People suppressed data for sample sizes of less than five. Therefore, attendance data were not provided when a school grade had fewer than five students.

### 2.7. Analysis

For each attendance date, the day of the week, month, week number, and day of the year were generated.

Attendance data were excluded for Grades 11, 12, and 13 (students repeating year 12), as they were not the target group for the School Lunch Project. Data for grade ‘Unknown’ were also excluded. At schools that did not provide lunches to all students, the grades that did not receive the lunches were excluded from the analyses. The same grades from the comparison school were also excluded. One grade in one School Lunch Project school only had attendance data for 10 days in 2022, as the rest of the year, there were fewer than five students. This grade was excluded from the analysis of that school and its comparison school.

Dates with very low attendance rates (<30%) were examined to determine if there was a reason for the low attendance. Data were excluded for three weeks in 2020 when overall attendance rates were low (week 12: 66.7%, week 13: 29.5%, week 14: 7.0%). This was at the start of the COVID-19 pandemic, and parents were advised to keep children at home (week 12), and then schools commenced learning from home for 30 to 40 days [27]. Data from the 9th of November 2022 were also excluded as it was a state-wide teacher stop work action day, and attendance was not consistently recorded across schools. Data from two individual schools were also excluded for one day each, where attendance was less than 40% for most of the grades.

### 2.8. Attendance on School Lunch Days versus Non-School Lunch Days

School Food Matters provided data for each school regarding the days the lunches were provided and the grades provided with lunch each day. For each grade at each school, a dichotomous school lunch day variable (school lunch day vs. non-school lunch day) was created for each date based on the day of the week and year. Linear mixed models were used to examine if attendance rates were different between school lunch days and non-school lunch days in the 17 consenting School Lunch Project schools during 2022 and 2023. This analysis followed the method described by Graubard and Korn [28] and the Stata command ‘mixed’. Linear mixed models were chosen as this analysis takes into account the nested groups of classes within schools and allows for covariates to be included in the models. Attendance rate was the continuous outcome variable; the dichotomous school lunch day variable (school lunch day vs. non-school lunch day) was the exposure variable, entered as a fixed effect; and school and grade were entered into the model as random effects. It was assumed that within-group errors had a first-order autoregressive structure (AR1). Day of year was used as the time variable. Covariates included in model 1 were day of the week (as attendance varied by day of the week), grade level (categorised as Kinder-Prep, Grades 1–5, Grades 6–7, Grades 8–10, as attendance rates decreased with increasing grade level), and week of year (as attendance varied throughout the year). Each year, there was a staggered start, with schools starting to provide lunches over the first four weeks of the term. Data from 23 May 2022 until the end of the year for 2022 and 6th March 2023 until 30 November 2023 were used for these analyses, as these were the weeks that all schools were providing the lunches.

Three sensitivity analyses were conducted while (1) excluding four small schools that showed a spike in attendance at 100% attendance, due to very small numbers of students per grade level in these schools; (2) excluding days with attendance rates less than 50%; (3) adding the number of days per week each grade received the lunches as a covariate in the adjusted model.

### 2.9. Attendance in School Lunch Project Schools versus Comparison Schools

Difference-in-difference regression was used to examine if there was a difference in the mean annual attendance rate between the School Lunch Project schools and comparison schools in 2023. We used the methods described by Donald and Lang [29] using the Stata command ‘didregress’. For this analysis, the outcome variable was the mean attendance rate for each grade at each school that was calculated for each year. Difference-in-difference analysis is the difference in attendance between the School Lunch Project and the comparison schools and the difference between ‘before’ intervention years and ‘after’ intervention years. This means it takes into account change over time in all schools before the school lunches were provided and estimates the ‘extra’ change in attendance that only occurs in the School Lunch Project schools in the years of the intervention (2022 and 2023) and not in the comparison schools (if there is an extra change). The estimated ‘average treatment effect in the treated’ is provided. The unadjusted model takes into account the group effects of school and effects of time (year). Model 1 makes additional adjustments for grade level.

To ensure the findings were not impacted by different school types (primary, secondary, and district), only School Lunch Project schools that had a comparison school of the same type were included in the analysis. School type, number of enrolments, and disadvantage level were used to select 11 School Lunch Project and 11 comparison schools with similar characteristics for the analysis. Three authors (KS, VD, and KJ) independently selected 11 School Lunch Project schools and 11 comparison schools of the same school type with similar number of enrolments and disadvantage level. The selected schools were discussed until there was unanimous agreement the most similar schools had been chosen.

All analyses were conducted using Stata/SE 18.0 for Mac, Revision 20 December 2023, StataCorp (College Station, TX, USA).

## 3. Results

### 3.1. Perceived Attendance

The online survey was completed by 65 staff from the 12 School Lunch Project schools that were selected for the evaluation. Increased attendance was reported as a benefit of the School Lunch Project by 22% (n = 14) of staff (Table 1).

A change in attendance was reported by 17% (n = 11) of staff, with 14% (n = 9) reporting an increase in attendance and two staff not reporting what the perceived change was (Table 1). Most school staff (69%) reported no change in attendance. The staff that reported a change in attendance and/or that increased attendance was a benefit of the lunch program were from 8 of the 12 schools.

A total of 37 teachers and support staff and 14 principals participated in an interview or focus group. Two principals from one school were interviewed due to changes in staff. In the open text responses to the survey and during discussions about attendance, a small number of staff reported attendance had increased for some students on the days the school lunches were provided.

*‘I know there are some families where attendance has been an issue, that they at least try to get their children to school on the day where there’s a hot lunch, that they might not be here any other day, but the hot lunch days they’ve been here for that meal’.* (Principal.)

*‘Attendance is better on lunch days’.* (Teacher.)

*‘Students that have a history of absenteeism are more likely to attend on the lunch program day’.* (Teacher.)

However, other staff reported no change in attendance or said that it was too soon to see these impacts.

*‘I was hoping attendance, academic performance, learning would improve but not yet’.* (Principal.)

*‘But our attendance is hard to tell at this stage’.* (Staff.)

### 3.2. Attendance on School Lunch Days versus Non-School Lunch Days

Of the 30 School Lunch Project schools, 17 (57%) provided consent for their attendance data to be provided to the research team, and 13 (43%) did not respond. The characteristics of these schools are shown in Table 2.

The majority of schools that consented were primary schools (65%), which reflects the high number of primary schools participating in the School Lunch Project. Eight schools commenced the lunch project in 2022, and nine commenced in 2023. During 2023, most schools provided lunches one to three days per week (mean 2.2 days per week). As some schools provided the lunches to different grades on different days, most students received the lunches one day per week during 2023 (mean 1.7 days per week). One school had a four-week cycle, where every grade received the lunches two days over the course of four weeks. The daily per cent attendance data for most School Lunch Project schools were normally distributed, with a mean daily attendance of 81.9% in 2022 and 82.9% in 2023.

Among the eight schools that commenced in 2022, mean attendance was nearly identical on school lunch days (80.6%) and non-school lunch days (80.8%, Table 3). During 2023, when all 17 schools provided the lunches, mean attendance was also similar on school lunch days (82.4%) and non-school lunch days (83.1%). In the linear mixed model, which takes into account the nested classes within schools and adjusts for day of the week, grade level and week number, mean attendance was similar between school lunch days and non-school lunch days in 2022 (difference 0.04, 95% CI −0.5, 0.6) and 2023 (difference 0.1, 95% CI: −0.2, 0.4, Table 3). Excluding the days when the attendance rate was less than 50% (N = 67 for 2022, N = 80 for 2023) and excluding the four small schools did not change the interpretation of the findings. Making additional adjustments for the number of days per week each grade received the lunches did not change the results.

### 3.3. Attendance in School Lunch Project Schools versus Comparison Schools

A total of 12 of the 30 (40%) invited comparison schools provided consent to this study. There were 11 School Lunch Project schools and 11 comparison schools of the same type (10 primary, 4 secondary, and 8 district schools) that were included in the analysis comparing attendance rates between School Lunch Project schools and comparison schools (six School Lunch Project and one comparison school were excluded). The characteristics of the schools are shown in Table 4. The number of students enrolled and level of disadvantage were similar between the School Lunch Project and comparison schools for each school type, with the exception of student enrolments for district schools, where the largest School Lunch Project school had nearly three times the enrolments of the largest comparison school. Of the School Lunch Project schools included in this analysis, seven started providing lunches in 2022, and four started in 2023. The lunches were provided one to four days per week. On the days the lunches were provided, all schools served all grades, with the exception of one school that provided the lunches three days per week, but Kinder students only received the lunches on one of these days (Kinder students only attended school three days per week).

**Figure 1:**
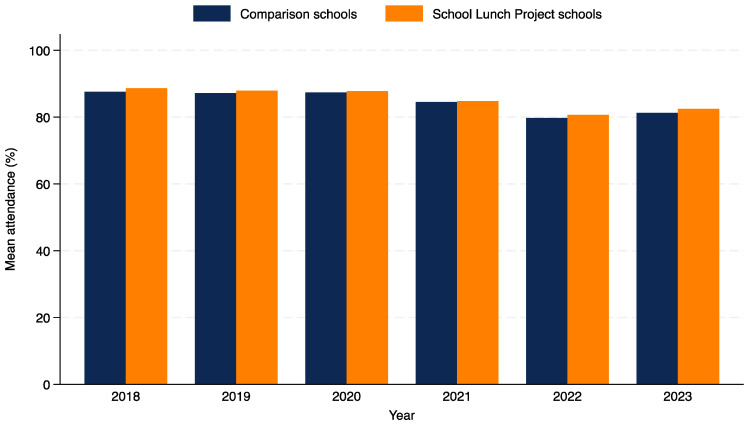
Mean per cent attendance for School Lunch Project and comparison schools, 2018–2023.

Figure 1 shows the mean attendance for the 11 School Lunch Project schools and 11 comparison schools during the four years prior to the School Lunch Project (2018–2021) and the two years of the School Lunch Project (2022–2023). Attendance rates were lowest in 2022, which is likely due to COVID-19. Tasmania is an island state, and to prevent the transmission of COVID-19, the borders were closed to non-residents in March 2020 for nearly 2 years. With the exception of an outbreak in March/April 2020 [30], there was no community transmission during this time. COVID-19 numbers peaked in Tasmania in 2022 as the island state’s borders re-opened for the first time post-pandemic in December 2021. High rates of community transmission meant students were not permitted to attend school for two weeks if they or a household member tested positive for COVID-19. They were also required to stay home if they had respiratory symptoms.

The average treatment effect of the School Lunch Project on student attendance was estimated, adjusting for grade, the group effect of school, and changes in attendance over time in all schools. There was no significant difference in the change in student attendance after the introduction of the School Lunch Project between the 11 School Lunch Project schools and the 11 comparison schools (average treatment effect in the treated: 1.2, 95% CI −0.7, 3.0, Table 5).

## 4. Discussion

This analysis of the Tasmanian School Lunch Project builds on previous work by using a mixed-methods approach to examine if the introduction of free nutritious cooked lunches, available to all students in the eligible grades, was associated with increased school attendance. A small number of school staff who participated in the survey and focus groups reported increased attendance on school lunch days, but most staff reported no change or considered it too early to identify this change. The school lunches were not associated with routinely collected (objectively measured) student attendance data. Attendance was similar on school lunch days and non-school lunch days among the 17 School Lunch Project schools. Overall attendance was also similar when compared to comparison schools that were not involved in the School Lunch Project.

Universal school lunches have been shown to improve food security, diet quality, and academic performance and have the potential to improve school attendance [3,12]. However, we found no association between school lunches and attendance. There are several possible reasons for this finding, including that school lunches do not impact school attendance and other strategies to increase attendance may be more beneficial. Our findings are consistent with studies that examined policy changes enabling universal free meals to all students in Kinder to Grade 3 in Scotland [11] and all students attending middle schools in New York City, USA [31].

Alternatively, the null findings may be explained by our inability to analyse specific groups of students. In the surveys and focus groups, a small number of school staff reported increased attendance among some students on the days the school lunches were provided. This is consistent with findings from the School Nutrition Project in the Northern Territory of Australia, where some key stakeholders reported increased attendance when meals (paid through Centrelink payments) were provided to 73 schools in remote and very remote communities [10]. No objectively measured attendance data were used in that study. It is possible that school lunches may improve attendance for some students, such as those experiencing food insecurity or greater levels of disadvantage. However, we were unable to specifically examine these groups of students as attendance data were provided for the grade level, not for individual students. Previous studies in New Zealand and the USA have shown that a universal school lunch program improved attendance among the most disadvantaged students but not the overall sample [8,9,32].

The null findings may also be explained by the low frequency of the lunches. Internationally, most school lunch programs provide lunch five days per week. The School Lunch Project was originally designed to provide lunches five days per week; however, due to budget and resource constraints, the lunches were provided 1–4 days per week (a mean of 2.4 days per week in 2022 and 2.2 days per week in 2023). Some schools scheduled the lunches for different grades on different days of the week. As a result, many students (from four schools in 2022 and 10 schools in 2023) only received the meals one day per week, which may be insufficient to noticeably improve attendance and to establish habituation, as some families reported forgetting which day the lunches were provided. Adjusting for the number of days each grade received the lunches did not affect the results when examining attendance on school lunch days and non-school lunch days. However, when comparing attendance between the School Lunch Project schools and the comparison schools, the sample size was not large enough to examine if attendance varied by the frequency of lunch provision.

It is also possible that the school lunches had not been provided for long enough, with only eight schools in this analysis providing the lunches for more than one year. Several staff said that they felt it was too soon to see any improvements in attendance. A study among elementary schools in the USA found universal free lunches were not associated with school attendance after the first year. However, during the second year, the number of students classified as having low attendance (missing at least 5% of school days annually) reduced by 3.5 percentage points compared to control schools [32]. In the New Zealand lunch trial, improved attendance was only observed after the students had received the daily lunches for three or four terms [9].

There are several limitations that should be taken into consideration when interpreting the findings. The School Lunch Project was optional for students in the eligible grades, with participation estimated to range between 65% and 90% across the schools. The provided attendance data would include students who were not participating in the School Lunch Project, which may underestimate or overestimate any potential effect. As the data were provided at the grade level, the difference-in-difference analysis does not account for the changing student cohorts in each grade each year. In addition, attendance data can be impacted by data quality issues. For example, if students were not marked as present or absent on a particular day, then they were not included in the denominator for the calculation of the attendance rate on that day. Attendance can be influenced by other factors not measured in this study, such as illness, family factors, COVID-19 restrictions, and initiatives introduced by the school to increase attendance. These factors should not differentially impact attendance on school lunch and non-school lunch days but could vary between the School Lunch Project and comparison schools. Many schools have informal systems in place to provide food to children in need, and the effect of the school lunches on attendance may be underestimated if the School Lunch Project schools provided informal lunches to students in need on days that were classified as non-school lunch days or if the comparison schools provided lunches to students in need. Our results may not be generalisable to schools outside Tasmania or with different socio-demographics.

The strengths of this study include the opportunity to evaluate a ‘real world’ intervention, the mixed-methods approach, which provided depth and breadth to the data collected, and the use of routinely collected (objectively measured) administrative data as an outcome measure. The use of difference-in-difference regression analysis is another strength, as it takes into account differences in attendance between the School Lunch Project schools and the comparison schools before the School Lunch Project commenced.

Future studies could identify the groups of students whose attendance may benefit the most from school lunches, such as those experiencing food insecurity or socioeconomic disadvantage. However, only providing lunches to these students is not recommended due to stigmatisation, and universal lunches have the potential to ensure all students have access to a nutritious meal. Universal free meals have also been shown to increase participation in school meal programs [3]. Although participation mostly increases among students who were previously not eligible to receive the meals, some studies have shown providing universal free meals can increase uptake among economically disadvantaged students [3,31]. Future research could also examine whether school lunches are associated with the type of absence. A higher proportion of unexcused absences (holiday, non-school related activity, or no reason given) has been shown to have a greater negative impact on academic outcomes than excused absences (such as illness or death in the family) [33].

## 5. Conclusions

The School Lunch Project provided the opportunity to investigate associations between providing nutritious cooked lunches to Tasmanian students and school attendance. While some staff reported that the lunches were associated with increased student attendance, this was not supported by objectively measured (routinely collected) attendance data. However, this study was not able to determine if there were differential associations for groups of students. In addition, school lunch programs may need to provide lunches more than one day per week to have an impact on student attendance. Further research is needed to explore whether school lunch frequency is associated with increased attendance.

## Figures and Tables

**Table 1:** Number (%) of staff (teachers, support staff, and principals) that reported increased attendance was a benefit of the School Lunch Project or they had noticed a change in attendance since the School Lunch Project had commenced.

	Yes		No		Unsure *	
Staff Role	n	%	n	%	n	%
Increased attendance was a benefit of the project						
Teacher	7	16.7	35	83.3	---	---
Support staff	5	27.8	13	72.2	---	---
Principal	2	40.0	3	60.0	---	---
Noticed a change in attendance						
Teacher	5	11.9	33	78.6	4	9.5
Support staff ^†^	4	23.5	9	52.9	4	23.5
Principal	2	40.0	2	40.0	1	20.0

* ‘Unsure’ was only a response option for the change in attendance question in the 2022 survey. ^†^ One support staff member did not answer the change in attendance question.

**Table 2:** Characteristics of the School Lunch Project schools that did and did not consent to the attendance analysis.

	Schools that Consented (N = 17)		Schools that Did Not Consent (N = 13)		Total (N = 30)	
Characteristic	n	%	N	%	n	%
School type						
Primary	11	64.7	6	46.2	17	56.7
Secondary	2	11.8	3	23.1	5	16.7
District	4	23.5	4	30.8	8	26.7
School disadvantage percentile *						
Mean (SD)	13.9	(10.5)	8.8	(6.6)	11.7	(9.3)
Year commenced School Lunch Project						
2022	8	47.1	7	53.8	15	50.0
2023	9	52.9	6	46.2	15	50.0
Number of days/week the lunches were provided during 2023						
1	5	29.4	7	53.8	12	40.0
2	6	35.5	3	23.1	9	30.0
3	5	29.4	3	23.1	8	26.7
4	1	5.9	0	0	1	3.3
Number of days/week each grade received the lunches during 2023						
0.25	1 ^†^	5.6	0	0	1	3.3
1	10	55.6	8	61.5	18	60.0
2	2	11.1	2	15.4	4	13.3
3	4 ^‡^	22.2	3	23.1	7	23.3
4	1	5.6	0	0	1	3.3

* Disadvantage was estimated using the Index of Community Socio-educational Advantage (ICSEA) percentile. ICSEA is calculated using student characteristics (parent’s occupation and parent’s education), the geographical location of the school, and the proportion of Indigenous students. A percentile of 100 indicates the most advantaged, and 0 indicates the most disadvantaged. The ICSEA percentiles for 2022 are presented here. ^†^ One school provided lunches to each grade two days every four weeks. ^‡^ One school provided lunches three days per week, but Kinder students only received the lunches on one of these days.

**Table 3:** Difference in mean per cent attendance on school lunch days and non-school lunch days for the 17 School Lunch Project schools, 2022 and 2023.

	Raw Data				Modelled Data			
Year of Attendance	Non-School Lunch Days		School Lunch Days		Unadjusted		Model 1 *	
	Mean	SD	Mean	SD	Diff	95% CI	Diff	95% CI
2022 (N = 6824) ^†^	80.8	11.3	80.6	10.2	−0.004	−0.5, 0.5	0.04	−0.5, 0.6
2023 (N = 15,596)	83.1	10.6	82.4	10.4	0.2	−0.1, 0.5	0.1	−0.2, 0.4

SD, standard deviation; Diff, difference in attendance rates between non-school lunch days and school lunch days, estimated using linear mixed models; CI, confidence interval. * Model 1 adjusted for grade level, day of the week, and week number. ^†^ N is the number of attendance values included in the analysis each year (attendance provided each day, for each grade, at each school).

**Table 4:** School type, number of enrolments, and level of disadvantage for the 11 School Lunch Project schools and 11 comparison schools.

		Number of Students Enrolled		Disadvantage Percentile *	
School Type	N	Mean	Range	Mean	Range
Primary schools					
School Lunch Project schools	5	239.2	142–325	11.2	5–17
Comparison schools	5	249.6	116–334	11.4	6–18
Secondary schools					
School Lunch Project schools	2	341.5	335–348	6.5	5–8
Comparison schools	2	398.5	351–446	7.5	7–8
District schools					
School Lunch Project schools	4	333.0	92–848	11.0	7–16
Comparison schools	4	200.8	142–291	11.8	9–15

* Disadvantage was estimated using the Index of Community Socio-educational Advantage (ICSEA) percentile. ICSEA is calculated using student characteristics (parent’s occupation and parent’s education), the geographical location of the school, and the proportion of Indigenous students. A percentile of 100 indicates the most advantaged, and 0 indicates the most disadvantaged. The ICSEA percentiles for 2022 are presented here.

**Table 5:** The average treatment effect of the school lunches on the mean per cent attendance in 11 School Lunch Project schools and 11 comparison schools.

Raw Data				Modelled Difference-in-Difference Estimate			
Comparison Schools		School Lunch Project Schools		Unadjusted		Model 1 *	
Mean	SD	Mean	SD	ATET	95% CI	ATET	95% CI
81.3	6.9	83.0	6.0	1.2	−0.7, 3.0	1.2	−0.7, 3.0

SD, standard deviation; ATET, estimated average treatment effect in the treated (School Lunch Project schools); CI, confidence interval. Mean (SD) is provided for 2023, when all the School Lunch Project schools provided lunches. However, the analysis uses the data from all 6 years (2018–2023). * Model 1 adjusted for group effects (school), time effects (year) and grade.

## Data Availability

Restrictions apply to the availability of these data. Data were obtained from the Department for Education, Children and Young People and are available from the authors with the permission of the Department for Education, Children and Young People.
